# Abstract of Proceedings of the Buffalo Medical Association

**Published:** 1859-07

**Authors:** W. H. Butler


ART. IV.—*Abstract of Proceedings of the Buffalo Medical Association.* Reported by W. H. Butler, M. D., Secretary.

Revaccination.—By Thos. F. Rochester, M. D. The unusual prevalence of variolous disease for the past six months, not only in this vicinity but also in other sections, awakens anxious inquiry as to the cause. That the boon to mankind of Jenner's inestimable discovery is as precious as ever, cannot be doubted by any medical observer; but that it has fallen into slight popular disrepute is unquestionable. This is due to several causes, which may be thus enumerated:

*First.* Neglect of vaccination. This is especially true of the offspring of foreigners, and results quite as often from parsimony as from ignorance or carelessness; incredible as it may seem, and criminal as is the advice, this neglect is often urged upon parents by many practitioners of transcendental and infinitesimal doctrines.

*Second.* The employment of improper virus, and negligence in discriminating between a true and a spurious result.

*Third.* Depreciation in the activity and protective power of vaccine, induced by long and uninterrupted transmission through human subjects.

*Fourth.* The frequent employment of vaccine crust or scale in place of vaccine lymph.

*Fifthly* and finally,—the well-established fact, that, from peculiar idiosyncrasy, especially marked in certain families and individuals, there is a proneness to variola, and hence a briefer limit than usual to the protection of vaccina.

These several propositions will be considered seriatim; and it is believed they can be sustained by conclusive evidence. The following, from the city physician, illustrates the large neglect of vaccination, and the comparative protection afforded by it when effected, as well as its loss of efficacy in certain cases.

BUFFALO, May 26, 1859.

DR. ROCHESTER, *Dear Sir*:—In compliance with your request I have examined my data since I have held the office of health-physician (from January 5th to May 1st, 1859). I had reported to the health-commissioners 284 cases of small-pox and varioloid. Deaths 27. Of these, 105 had been vaccinated—or more than one-third; they were nearly all vaccinated in Germany when about one year old, by a governmental requisition. They generally had varioloid of either very slight or of moderate severity; though the exceptions were numerous, and among those exceptional cases, six had aggravated small-pox and died of the disease. I believe these facts cover all your inquiries. I intend to embody them more at length, with others, in a report at the close of my term of service; but so far as they go they are cheerfully submitted to your disposal.

And I remain yours respectfully,

P. H. STRONG.

From the above kindly furnished communication we find—A large number of unvaccinated persons; and of these, out of 179 cases there were 21 deaths, or nearly one in nine. Of those who had been vaccinated, 105 in all, there were six deaths, or one in seventeen. We have here a statistical demonstration, of what every physician is cognizant, to wit, criminal carelessness on the part of parents, in neglecting to protect their offspring.

As to the use of improper virus, and dereliction of duty on the part of the medical attendant in discriminating between a true and spurious result,—your reporter has seen a yellow herpetic crust employed, and has repeatedly known a violently inflamed arm of phlegmonous character regarded as demonstrative proof of success; and that by practitioners of good standing. It may not, perhaps, be out of place to state in this connection, the characteristic local evidences of true vaccina. A papule should be developed not sooner than the end of the third, and not later than the termination of the fifth day after the introduction of the virus. Twenty four hours after the appearance of the papule, it should be metamorphosed into a vesicle. The vesicle should be fully formed and umbilicated by the seventh day. From the eighth to the ninth day, it is converted into a pustule, and is surrounded by an areola of greater or less extent; the redness should always be circular and with defined edges. By the tenth day the areola should fade, and incrustation be established in the pustule. From the twelfth to the sixteenth day the crust hardens, dries, and separates—and should leave a depressed or pitted *circular* excavation in the skin. The crust, when dry, should be of a dark mahogany color, circular with a slight central depression, and of a firm, not friable texture. The size of the crust may vary from one-fourth to one-half an inch in diameter. Where they are larger, there is apt to be too much local inflammation. Your reporter is informed by Dr. C. H. Wilcox, that "in a majority of his cases of varioloid, most of the patients had from two to three very large vaccine cicatrices, such as are usually seen on German emigrants; and that he ascribes the want of complete protection, to the excessively suppurative character of the vaccination."

Respecting the third proposition,—that vaccine deteriorates by prolonged transmission through the human race, there seems to be strong, but not absolutely positive evidence. Jenner feared that such would be the result. Watson, who was a member of the London Vaccine Board for one year, thinks he saw, to all appearance, as good an effect as possible produced by virus transmitted from person to person directly from Jenner's time; he states, however, that such is not the opinion of Simon, of Brisset, of Meyer, of Estlin, and Gregory; who suppose that the vaccine, in passing through the bodies of many persons, becomes enfeebled and, although it may produce its characteristic local effects, loses measurably its protective property. This seems to be demonstrated by the records of the Prussian army, in which revaccination is always effected with the new levies. In 1833, it succeeded in 33 out of 100, and has gone on with an increasing ratio up to 1857, when the proportion was 70 in 100. The vaccine employed was not taken from the cow, but transmitted from individual to individual. With such strong grounds for suspicion of efficacy, it seems no less than a duty, to obtain, occasionally at least, virus from the animal. Among the responses to certain questions proposed by the French Academy, relating to vaccination, the following is cited by Condin. "The preservative power of vaccine matter does not seem to be intimately connected with the intensity of the symptoms of vaccination; nevertheless it is prudent to *regenerate* vaccine matter as frequently as possible, to preserve its protective power."

The *fourth* proposition,—that vaccination effected from the crust or scale is inferior to that from lymph, has been frequently urged upon this association by the writer. For proof of this he refers to the subjoined table. It will be seen that in several instances lymph succeeded after the crust had been tried and had failed. The writer embraces this opportunity to correct an error in the minutes of the Association, in which, at a previous meeting, he is represented to have said that vaccination from lymph was effectual for life; he only stated, that he thought it much more reliable than that from the crust. The only objection that can be urged against lymph, is its perishable nature, which entails the necessity of very frequent renewal. Taken upon quills and confined in glass, it cannot be relied upon for more than ten days in warm weather. In winter I have often employed it sucessfully after twice that period. Lymph is used exclusively in the dispensaries in New York, also in Paris and London. Watson says, of Mr. Marson, the resident surgeon of the London Small-pox and Vaccination Hospital, "This gentleman has vaccinated between forty and fifty thousand persons," and he (Marson) asserts that, "one of the principal causes of failure in vaccinating, and of subsequent insecurity of the individual, even when the vaccination *does take effect*, is the want of care in the selection of the lymph; lymph for use is in its best state on the day week from the vaccination. It should be taken when the vesicles are plump, and just before the formation of the areola. Under no circumstances should it be taken for use later than twenty-four hours after the areola has begun to form." Besides the greater security afforded by lymph, it is more easily and rapidly inserted than the crust, is often done without waking the infant, and while it almost invariably "takes," the writer has never known it to give rise to the severe erysipelatous inflammation, that he has in several instances seen follow the introduction of the crust. It is also more rapid in its course than the crust, usually by at least twenty-four hours, a consideration not to be lost sight of after exposure to variola. Without urging the consideration of *this matter* (as the Autocrat would say) further upon the members of the Association,—

I now pass to the fifth and last proposition, the proneness of certain individuals to variola. The writer is cognizant of a case, where a man twice passed through the disease with safety, and succumbed to a third attack. He has also seen two persons suffering for the second time with the affection; both had it lightly and recovered. He has also seen four cases of successful vaccination after variola. It is generally supposed that those persons who are not susceptible of vaccination are exempt from variola. This is probably the rule; but it has exceptions. A gentleman residing in this city, had been repeatedly vaccinated from infancy up, without effect; he contracted some variola in his thirtieth year, during an epidemic, and just twenty days after a careful but unsuccessful attempt at vaccination (made however with crust). He escaped with his life, deeply scarred and pitted. From the middle of December, 1858, to the 1st of May, 1859, the writer revaccinated one hundred and thirty-seven persons of all ages and conditions. With a portion of these he was compelled to use the vaccine crust, being unprovided with lymph. Some of these he afterwards tested with lymph; and in a few instances, where the crust had failed, lymph succeeded. It is much to be regretted that no opportunity was afforded of trying lymph in *all* the failures with crust.

A revaccination, even when successful, does not always pursue the regular course of a primary vaccination. It is sometimes more rapid and sometimes more slow in its operation. In the table, no case is claimed as successful (marked P., *i. e.* perfect) where the arm was sore only a few days, and where there was not formed, the characteristic, round, dark, firm crust, imbedded in a cup-like excavation. It is not claimed that successful revaccination is positive evidence of inefficacy of primary vaccination ; but as revaccination is the only feasible test of protection against variola, it is probable that most of those susceptible to vaccinia are equally so to variolous disease. Appended to this paper is a statistical record of the one hundred and thirty-seven revaccinations above cited. A summary of the same gives sixty-seven successful revaccinations, within a fraction of one-half of the whole number ; these are marked P. (perfect.) The proportion is larger than the writer supposed, but much less than the ratio in the Prussian army, which it will be remembered is seventy in every hundred. An analysis of the record shows the largest proportion of successful revaccinations in persons aged from three to sixteen years, inclusive, there being of this class thirty-three, of whom twenty-two, or two-thirds, were susceptible. This certainly gives good ground for the suspicion of vaccine deterioration. The next largest proportion is found in persons aged from thirty-six to seventy years. Of these there were twenty-seven, of whom thirteen, or about one-half, were revaccinated with effect. Of those, from seventeen to thirty-six years old, there were seventy-seven; revaccination was successful with thirty-two, or something less than one half. Of the whole number of cases, twenty-two were doubtful or imperfect ; these are marked I. In forty-eight, there was either no result or but slight soreness ; these are marked N. The six is not mentioned, as immaterial. No record was kept of the appearance of the primary vaccine scar ; but it was observed that whether slight or marked, or whether one or more, no safe inference could be drawn therefrom. Vaccine *crust* was used in twenty-seven cases ; it *took* but in three. Four of the twenty-seven were afterwards revaccinated with *lymph*,—three of them successfully. One hundred and ten were revaccinated with lymph only ; with sixty-one, or more than one-half, it was effectual.

### TABULAR STATEMENT

### OF ONE HUNDRED AND THIRTY-SEVEN REVACCINATIONS.

| Age when vaccinated. | Age when revaccinated. | Lymph or Crust used. | Result. | Age when vaccinated. | Age when revaccinated. | Lymph or Crust used. | Result. |
|---|---|---|---|---|---|---|---|
| 12 | 22 | C. | N. | 1 | 16 | L. | P. |
| 6 | 21 | C. | I. | 5 | 22 | L. | P. |
| 7 | 20 | C. | N. | 1 | 24 | L. | I. |
| 1 | 23 | L. and C. | P.* | 8 | 20 | L. | P. |
| 25 | 36 | C. | I.† | 5 | 22 | L. | N. |
| 10 | 23 | L. | P. | 1 | 11 | L. | P. |
| 10 | 21 | C. | N. | 1 | 14 | L. | P. |
| 10 | 29 | C. | N. | 1 | 8 | L. | P. |
| 1 | 25 | C. | N. | 1 | 25 | L. | N. |
| 8 | 22 | C. | I. | 4 | 20 | L. | P. |
| 4 | 21 | C. | N. | 1 | 58 | L. | N. |
| 4 | 24 | C. | N. | 1 | 45 | L. and C. | P.§ |
| 10 | 20 | C. | I. | 1 | 19 | L. | P. |
| 4 | 19 | C. | N. | 1 | 16 | L. | N. |
| 1 | 24 | C. | N. | 1 | 11 | L. | N. |
| 1 | 19 | L. and C. | P.* | 1 | 14 | L. | P. |
| 1 | 38 | L. and C. | N.‡ | 1 | 48 | L. | N. |
| 12 | 25 | C. | N. | 1 | 35 | L. | P. |
| 7 | 20 | C. | P. | 10 | 70 | L. | P. |
| 7 | 23 | C. | P. | 5 | 50 | L. | P. |
| 1 | 32 | C. | N. | 3 | 40 | L. | N. |
| 1 | 22 | C. | N. | 1 | 30 | L. | I. |
| 5 | 23 | C. | N. | 1 | 9 | L. | P. |
| 14 | 21 | C. | I. | 1 | 22 | L. | P. |
| 1 | 25 | C. | N. | 3 | 30 | L. | I. |
| 15 | 23 | L. | P. | 5 | 28 | L. | N. |
| 1 | 21 | L. | P. | 7 | 42 | L. | P. |
| 1 | 30 | L. | P. | 1 | 20 | L. | N. |
| 2 | 32 | L. | N. | 1 | 40 | L. | P. |
| 28 | 40 | L. | N. | 1 | 11 | L. | P. |
| 1 | 25 | L. | P. | 4 | 36 | L. | N. |
| 1 | 30 | L. | I. | 1 | 9 | L. | I. |
| 2 | 40 | L. | I. | 4 | 40 | L. | P. |
| 1 | 12 | L. | P. | 1 | 12 | L. | P. |
| 1 | 16 | L. | P. | 1 | 19 | L. | P. |
| 1 | 30 | L. | P. | 1 | 11 | L. | I. |
| 1 | 58 | L. | P. | 1 | 7 | L. | N. |
| 1 | 19 | L. | N. | 4 | 35 | L. | I. |

* Crust failed; Lymph succeeded. † Never successfully vaccinated.
‡ Both failed. § Crust failed.

TABULAR STATEMENT—Continued.

| Age when vaccinated. | Age when revaccinated. | Lymph or Crust used. | Result. | Age when vaccinated. | Age when revaccinated. | Lymph or Crust used. | Result. |
|---|---|---|---|---|---|---|---|
| 9 | 37 | L. | N. | 1 | 28 | L. | I. |
| 1 | 24 | L. | P. | 1 | 25 | L. | P. |
| 1 | 14 | L. | P. | 1 | 28 | L. | N. |
| 5 | 60 | L. | P. | 1 | 24 | L. | I. |
| 10 | 65 | L. | P. | 1 | 20 | L. | N. |
| 1 | 20 | L. | I. | 1 | 20 | L. | N. |
| 1 | 17 | L. | P. | 1 | 29 | L. | P. |
| 1 | 25 | L. | P.* | 1 | 27 | L. | I. |
| 1 | 24 | L. | P. | 5 | 30 | L. | N. |
| 1 | 30 | L. | P. | 1 | 5 | L. | N. |
| 1 | 16 | L. | P. | 1 | 3 | L. | P. |
| 1 | 14 | L. | N. | 1 | 18 | L. | N. |
| 1 | 12 | L. | P. | 3 | 53 | L. | P.‡ |
| 1 | 9 | L. | N. | 3 | 43 | L. | P.‡ |
| 1 | 7 | L. | N. | 2 | 21 | L. | P.‡ |
| 5 | 62 | L. | N. | 2 | 16 | L. | P.‡ |
| 1 | 35 | L. | I. | 2 | 11 | L. | P.‡ |
| 1 | 20 | L. | P. | 1 | 5 | L. | P.‡ |
| 1 | 18 | L. | P. | 1 | 38 | L. | I. |
| 1 | 16 | L. | N. | 1 | 9 | L. | P. |
| 1 | 12 | L. | P. | 1 | 36 | L. | P. |
| 1 | 22 | L. | N. | 1 | 35 | L. | P. |
| 1 | 20 | L. | I. | 1 | 45 | L. | I. |
| 1 | 20 | L. | N. | 1 | 14 | L. | P. |
| 1 | 40 | L. | P.† | 1 | 11 | L. | P. |
| 1 | 45 | L. | N. | 1 | 12 | C. | P. |
| 1 | 24 | L. | N. | 4 | 25 | L. | I. |
| 1 | 20 | L. | P. | 1 | 17 | L. | P. |
| 1 | 16 | L. | P. | 1 | 60 | L. | N. |
| 1 | 19 | L. | N. | 1 | 30 | L. | P. |
| 1 | 60 | L. | N. | | | | |

Dr. Rankin reported the following case :

Tuesday, May 3d, 6 P. M., was called to attend P. L., aged 23, of temperate habits, strong and robust to a remarkable degree; who had been kicked by a horse. Found my patient lying upon a bed, laboring under an attack of tetanus,—*all* the symptoms developed, with the exception of *trismus.* The face was distorted, with the upper lip curled up, hard and unyielding, underneath the nostril; eyes turned up far back into their sockets; pulse over a hundred, full and hard, with a cold and profuse perspiration covering the face and trunk. The spasms extended to the muscles

* Roseola vaccinia appeared on the whole body on the fourth day.

† Had repeatedly been vaccinated before with crust, without success.

‡ All in one family.

of the trunk, the larger muscles of the extremities, and the hands and fingers ; the muscles of the tongue and jaw not being affected,—so that he could swallow, though he did not speak until after the paroxysms passed off. The three great characteristics of tetanus were developed—opisthotonos, emprosthotonos, and pleurosthotonos ; though the latter not so complete as the former ones.

All at once he would be drawn into such a position that his head and heels alone, resting upon the bed, supported him, while his body would be curved back, describing a complete arch. In an instant, almost with the quickness of thought, the opposite posture would be assumed ; his head and feet nearly meeting, but his knees not flexing, the legs remaining stiff. The lateral bending was not so marked, being but slight.

These paroxysms were terrible to behold, lasting nearly eight hours ; there being no interval—each succeeding the other.

My friend Dr. L. P. Dayton, was called in consultation. Our prognosis was that the patient would die ; and we could but endeavor to palliate the symptoms, there being no hope of cutting them off. Not having any chloroform, a solution of antimon. tart. gr. ii., morphia, gr. ii., in four teaspoonfuls of water, was prepared ; one teaspoonful of this prescription to be given every half-hour. We also took about three pints of blood from the arm.

I afterwards sent for chloroform ; but it seemed to have no effect, and was abandoned. I left about eight o'clock, P. M. On returning at midnight, found my patient a little more quiet ; the spasms still continued, but not with so great violence. Continued the same prescription (antimon. tart. and morphia), giving it at the same intervals, with the addition of brandy. Near two o'clock, A. M., the convulsions succumbed to this treatment, and he went to sleep ; remaining so till daylight.

On awaking, he was delirious ; remaining so for two days. This latter symptom, after the application of a large blister to the nape of the neck, extending down between the shoulders, disappeared. In a few days he was about the streets, as well as ever.

*Remarks.*—The interest of this case is the symptoms developed, and what caused them. On questioning him, after convalescence, he stated that the horse struck him but once, and that blow was immediately over the heart. Remembered receiving the blow, which knocked him down. Thinks he did not strike upon his head or back, but more in a sitting posture. Immediately got up, and walked out of the stable into the yard. Asked one of his companions to go for me. Beginning to feel a dizziness come over him, he fell to the ground in a convulsion. This is all he remembers.

No abrasion was found, nor even a bruise, upon his body. Then, what caused all these symptoms described? We could account for them in only one way; that being, a shock to the sympathetic system. Watson says that the causes of tetanus are very obscure, and admit of much speculation.

*Cancerous Tumor simulating Thoracic Aneurism.* Reported for Prof. Rochester, by A. J. Steele, M. D., Resident Physician of Buffalo General Hospital.

W. G., aet. 32, laborer, nat. Ireland, admitted May 6th, 1859.

*History:* Has been in this country ten years, habits temperate, married, has had two children, both dead, no hereditary tendency to disease. Five years ago had ague. Has worked for the Water Works Co. ever since. "Took cold" about six months ago; had severe pain in chest and back; noticed a swelling in the epigastric region, with local tenderness; the swelling and tenderness diminished, but never entirely ceased. As the swelling diminished below, pain became severe in the back and in the upper part of the chest; and, corresponding with the seat of the pain in front, a tumor appeared, at first small, but gradually enlarging; subject, however, to alterations in size. Coughs a good deal, especially on lying down; but does not expectorate. Has lost considerable flesh. Surface cool and pale; pulse feeble and irregular, ranging from 80 to 100.

May 7th, examined by Dr. Rochester. A tumor, three inches broad by two in length, occupies the upper sternal region; it is bulging, prominent, and conoidal; the skin covering it is natural in appearance, but for some distance below the tumor the superficial veins are very prominent. Palpation discovers elasticity and crepitation, but no fluctuation. Pulsation is detected by auscultation, by sight, and touch. There is occasionally, but by no means constantly, a slight purring thrill; bellows murmur constant in both subclavian regions, not heard over the body of the tumor. Heart sounds—first, indistinct; second, sharp. Apex impulse, an inch and a half below left nipple, and toward the medium line, very feeble. Percussion shows dullness over the whole sternal region, and over the whole of the right thorax, both in front and behind. Auscultation detects faint bronchial respiration, mucous râles, and feeble vesicular murmur. Percussion resonance good over left thorax, with, to the ear, strong vesicular murmur, and occasional sibilant and mucous râles.

*Diagnosis:* Mediastinal tumor pressing upon the right bronchus, and producing engorgement of right lung; the tumor is either traversed by a large artery or is purely aneurismal, probably the latter; the softening of the sternum, due to absorption induced by pressure. Treatment palliative and sustaining.

May 24th. No particular change until this time; when he was attacked with severe dyspnea, and became quickly comatose. He was aroused with difficulty, and rallied under the free administration of stimulants. Coincident with the relief from the alarming dyspnea, a second tumor was observed, occupying the middle sternal region, and separated from the upper tumor by a narrow sulcus; the sternum yielding, and thus abating the suffocative pressure.

From this time forward G. could not lie down, nor could he swallow solid food. He was not, however, materially worse than before; in fact, he was in better spirits, and thought he was improving.

June 4th, 1 A. M. He left his bed to get a drink of water, and remarked to a fellow-patient that his cough was troublesome, for which he took morphine, gr. $\frac{1}{8}$th; he was soon after observed to be breathing heavily; was found to be perfectly comatose, and died at 4 A. M.

Postmortem examination disclosed an encephaloid mass, four inches long, by two in diameter, embracing a portion of the aortic arch, and surrounding the right bronchus and lower portion of the trachea. The right pleura was three-fourths filled with straw-colored serum, and the lung, which was forced directly upward, and held firmly by pleuritic adhesions, was infiltrated with pasty purulent matter (cancerous?). The left lung was crepitant, but adherent on all sides by old exudations. The bronchial glands were much enlarged, and filled with cerebriform deposit. The sternum was much softened, and its ossific structure nearly all destroyed, a few spiculæ only being left. The portions corresponding with the tumors, were sacculated, but contained at the examination a little serum only. Heart and large vessels normal. The morbid specimens are submitted to the members of the Association for examination.

